# Marker Aided Incorporation of *Saltol*, a Major QTL Associated with Seedling Stage Salt Tolerance, into *Oryza sativa* ‘Pusa Basmati 1121’

**DOI:** 10.3389/fpls.2017.00041

**Published:** 2017-01-26

**Authors:** N. Naresh Babu, S. Gopala Krishnan, K. K. Vinod, S. L. Krishnamurthy, Vivek K. Singh, Madan P. Singh, Renu Singh, Ranjith K. Ellur, Vandna Rai, Haritha Bollinedi, Prolay K. Bhowmick, Ashutosh K. Yadav, Mariappan Nagarajan, Nagendra K. Singh, Kumble V. Prabhu, Ashok K. Singh

**Affiliations:** ^1^Division of Genetics, ICAR – Indian Agricultural Research InstituteNew Delhi, India; ^2^ICAR – Indian Agricultural Research Institute, Rice Breeding and Genetics Research CentreAduthurai, India; ^3^ICAR – Central Soil Salinity Research InstituteKarnal, India; ^4^Division of Plant Physiology, ICAR – Indian Agricultural Research InstituteNew Delhi, India; ^5^ICAR – National Research Centre on Plant BiotechnologyNew Delhi, India

**Keywords:** *Saltol*, salinity tolerance, marker assisted backcross breeding, foreground selection, grain and cooking quality, expression profiling, *OsHKT1;5* gene, Basmati rice

## Abstract

Pusa Basmati 1121 (PB1121), an elite Basmati rice cultivar is vulnerable to salinity at seedling stage. A study was undertaken to impart seedling-stage salt tolerance into PB1121 by transferring a quantitative trait locus (QTL), *Saltol*, using FL478 as donor, through marker assisted backcrossing. Sequence tagged microsatellite site (STMS) marker RM 3412, tightly linked to *Saltol* was used for foreground selection. Background recovery was estimated using 90 genome-wide STMS markers. Systematic phenotypic selection helped in accelerated recovery of recurrent parent phenome (RPP). A set of 51 BC_3_F_2_ lines homozygous for *Saltol* were advanced to develop four improved near isogenic lines (NILs) of PB1121 with seedling stage salt tolerance. The background genome recovery in the NILs ranged from 93.3 to 99.4%. The improved NILs were either similar or better than the recurrent parent PB1121 for yield, grain and cooking quality and duration. Biochemical analyses revealed significant variation in shoot and root Na^+^ and K^+^ concentrations. Correlation between shoot and root Na^+^ concentration was stronger than that between root and shoot K^+^ concentration. The effect of QTL integration into the NILs was studied through expression profiling of *OsHKT1;5*, one of the genes present in the *Saltol* region. The NILs had significantly higher *OsHKT1;5* expression than the recurrent parent PB1121, but lower than FL478 on salt exposure validating the successful introgression of *Saltol* in the NILs. This was also confirmed under agronomic evaluation, wherein the NILs showed greater salt tolerance at seedling stage. One of the NILs, Pusa1734-8-3-3 (NIL3) showed comparable yield and cooking quality to the recurrent parent PB1121, with high field level seedling stage salinity tolerance and shorter duration. This is the first report of successful introgression of *Saltol* into a Basmati rice cultivar.

## Introduction

Basmati rice from the Indian sub-continent is acclaimed world over for its exquisite grain quality especially for its superior eating quality combined with pleasing aroma. Pusa Basmati 1121 (PB1121) is a popular Basmati rice variety, bred by the ICAR-Indian Agricultural Research Institute (ICAR-IARI), New Delhi. PB1121 commands a premium position in the national and international market over other Basmati rice varieties because of its excellent grain quality (Ellur et al., 2016a). It now occupies over 60% of the total Indian Basmati rice area (Ellur et al., 2016b) covered under geographical indication (GI) notified states of Punjab, Haryana, Himachal Pradesh, Delhi, Uttarakhand, parts of Western Uttar Pradesh and Jammu and Kashmir (GIR, 2010). In the state of Haryana alone, more than 50% of the Basmati rice area is covered by PB1121 (APEDA, 2014), where more than 21% of agricultural land is salinity affected (Bhumbla, 1978) most of which is irrigated low lands. Inland salinity is emerging as one of the major production constraints in Basmati growing regions of Indian subcontinent primarily due to faulty irrigation practices and injudicious use of chemical fertilizers. Salinity poses a serious impediment to the extensive cultivation of PB1121 in states of Haryana and the neighboring areas in the state of Uttar Pradesh. The yield potential of PB1121, grown on approximately 1.35 million hectares of Basmati area in India is not fully exploited in salt affected soils due to its susceptibility to seedling stage salinity stress. Therefore, incorporation of seedling stage salinity tolerance in PB1121 can help to improve its adaptation to salt affected soils and in sustaining productivity.

Rice plants are highly salt sensitive at seedling (Khan et al., 1997), panicle initiation and pollination stages (Khatun and Flowers, 1995; Zeng et al., 2001), resulting in poor crop establishment. Salt stress at reproductive stage affects grain formation and grain quality. In rice, enormous variability has been observed for salt tolerance, which makes genetic improvement of salt tolerance a possibility (Flowers and Yeo, 1981; Akbar et al., 1986; Platten et al., 2013), but progress in salt tolerance breeding through conventional means has been painfully slow. Salt tolerance itself is a complex trait to measure (Munns, 2002) often accompanied with ambiguity created by high environmental influence and the phenotypic screening demands huge labor, space and laborious experiments (Flowers and Yeo, 1997; Gregorio et al., 2002; Yamaguchi and Blumwald, 2005; Ismail et al., 2007; Thomson et al., 2010). Molecular marker technologies have enabled mapping of specific genomic regions linked to salt tolerance which can accelerate the development of improved varieties though marker assisted selection (MAS, Vinod et al., 2013). Success of molecular marker assisted breeding in rice (Gopalakrishnan et al., 2008; Singh et al., 2011, 2013; Singh A. et al., 2012; Singh V.K. et al., 2012; Khanna et al., 2015; Ellur et al., 2016a,b) and in several other crops have unequivocally proved the advantage of MAS in enhancing the efficiency and accuracy of breeding improved varieties with resilience to biotic and abiotic stresses (Alpuerto et al., 2008).

Several salt tolerance-related quantitative trait locus (QTLs) associated with parameters such as Na^+^ and K^+^ ion uptake, ionic concentration and Na^+^/K^+^ ratio has been reported in rice (Koyama et al., 2001; Lin et al., 2004; Singh et al., 2007; Ammar et al., 2009; Sabouri et al., 2009; Haq et al., 2010; Pandit et al., 2010). A major QTL, *Saltol*, associated with seedling-stage salt tolerance and Na^+^/K^-^ ratio explaining 43–70% of phenotypic variation was mapped from a cross IR29/ Pokkali on chromosome 1 in rice (Gregorio et al., 1997; Bonilla et al., 2002). A seedling-stage salt tolerant recombinant inbred line of this cross, IR 66946-3R-178-1-1 (FL478), was commonly used as a donor for salt tolerance in rice. Haplotype analysis of the *Saltol* region of FL478 revealed a <1 Mb Pokkali chromosomal fragment located at 10.6–11.5 Mb region (Kim et al., 2009), containing *Shoot K^+^ content 1* (*SKC1*) and other Pokkali derived loci (Thomson et al., 2010). *SKC1* was originally mapped as a QTL, *qSKC1* (Lin et al., 2004) which was later fine-mapped and cloned to identify the gene *OsHKT1;5* encoding for a sodium transporter which regulates K^+^ homeostasis (Ren et al., 2005). This gene belongs to a large family of high affinity K^+^ transporters (HKT), and the sub-family *OsHKT1*, with five gene members (Platten et al., 2006; Vinod et al., 2013). *OsHKT1* genes are distinct from other HKT genes in their activity as Na^+^ uniporters, among which *OsHKT1;5* is identified to code for a transporter that is preferentially expressed in root xylem parenchyma and unloads Na^+^ from the xylem vessels. The salt tolerance of the rice landraces, Pokkali and Nona Bokra are hypothesized to be driven by *OsHKT1;5* gene (Ren et al., 2005).

A set of key *Saltol* linked markers, AP 3206, RM 8094 and RM 3412 have been identified for MAS for salt tolerance (Thomson et al., 2010; Aliyu et al., 2011). Although, there are reports of introgression of *Saltol* into rice varieties like BR11, BRRI Dhan 28, IR64, and AS996 (Huyen et al., 2012; Gregorio et al., 2013; Guo and Ye, 2014; Hasan et al., 2015), till date there are no reported attempt in Basmati rice. Transfer of *Saltol* into Basmati rice is challenging because the use of non-Basmati donor such as FL478 which may potentially impair Basmati quality. Therefore, marker assisted introgression of *Saltol* was carried out into the most popular Basmati rice variety, PB1121, by effectively combining phenotypic and molecular aided foreground and background selection to develop PB1121 near isogenic lines (NILs) with salt tolerance which was further validated by expression profiling.

## Materials and Methods

### Plant Materials

Because of its seedling stage salt susceptibility, PB1121, the most popular Basmati rice variety in India was used as the recurrent parent, and crossed with FL478 as the donor for *Saltol* in the study. PB1121 is the most popular Basmati rice variety of India, with exceptionally high cooked kernel elongation, high volume expansion besides possessing all other desirable grain and cooking qualities. However, owing to its susceptibility to soil salinity, PB1121 cannot be grown above medium salinity levels (>2.0 dS/m). FL478 is a non-aromatic genotype with coarse grains and characteristic red pericarp, which possesses seedling-stage salt tolerance up to 18 dS/m (Thomson et al., 2010) and exhibits good tillering under salt stress. The backcross derived lines from the cross PB1121/FL478//PB1121^∗^3 were designated with the prefix Pusa 1734.

### Molecular Marker Analyses

The extraction of total genomic DNA from the leaf tissues was carried out using Cetyl Trimethyl Ammonium Bromide (CTAB) method (Doyle, 1991). Foreground selection was done using microsatellite marker RM3412, the peak marker for *Saltol* QTL. The sequence tagged microsatellite site (STMS) markers namely RM35, RM1287, RM8094, RM10720, RM10748, and RM493 flanking the *Saltol* QTL on carrier chromosome (Chromosome 1) were used for recombinant selection. A set of 600 STMS markers distributed uniformly across the rice genome (Singh et al., 2010) were used for background selection. Polymerase chain reaction (PCR) was done in a PCR thermocycler (G-Storm, Somerset, UK). The 10 μl of the reaction mix was constituted with 30 ng of template DNA, 5 pmol each of both forward and reverse primers (Sigma, Inc.), 0.2 mM dNTPs (MBI, Fermentas), 1.5 mM of MgCl_2_ and 0.5 U of Taq polymerase (Bangalore Genei). The PCR constituted initial 5 min denaturation at 95°C, followed by 35 cycles of 30 s denaturation at 95°C, 30 s annealing at 55°C and 1 min extension at 72°C. After 35 cycles, the final extension was done for 7 min at 72°C and the products were cooled to 4°C. The amplicons were size separated through electrophoresis in 3.5% Metaphor^TM^ agarose gel (Lonza) mixed with 0.1 mg/ml of ethidium bromide. A 50 bp ladder DNA (MBI, Fermentas) was used as standard for visualizing and documenting the amplicons under trans-illumination in a gel documentation system (BioRad, USA).

### Marker Assisted Development of Improved Lines

Marker-assisted foreground selection was used for identifying lines heterozygous for the *Saltol*, in BC_1_F_1_ stage and homozygous lines from BC_3_F_2_ onward. To minimize donor segments on the carrier chromosome, flanking markers were used for recombinant selection. In the background selection, recurrent parent genome (RPG) recovery was analyzed with the help of polymorphic markers between PB1121 and FL478, distributed uniformly across the rice genome. The flanking markers used for recombinant selection were excluded from RPG recovery estimation as they could overestimate the genome recovery. Systematic selection for agro-morphological, grain and cooking quality traits was augmented with genotypic selection at every selection stage to ensure maximum RPP recovery.

A single BC_1_F_1_ plant heterozygous for *Saltol* linked marker RM3412 and highest RPG as well as phenotypic resemblance to PB1121 was backcrossed to develop the BC_2_F_1_ plants, which were subjected to foreground and background selection as well as phenotypic selection to select most desirable plant heterozygous for *Saltol* and with maximum recovery of RPG. The selected plants were further backcrossed to PB1121 to develop BC_3_F_1_s. Foreground, background and phenotypic selection cycle was repeated in BC_3_F_1_ as well, to recover plants heterozygous for *Saltol* and higher RPG recovery. The BC_3_F_2_ population was generated by selfing the selected plants, from which plants homozygous for *Saltol* were identified and advanced till BC_3_F_4_ generation. Further selection in subsequent generations was restricted only for agro-morphological, grain and cooking quality traits. RPG recovery was assessed at every step using genome wide STMS markers and was estimated with help of Graphical GenoTypes (GGT) Version 2.0 software (Van Berloo, 1999).

### Screening for Seedling Stage Salt Tolerance

The *Saltol* homozygous BC_3_F_3_ plants and the parents were evaluated hydroponically for seedling stage salt tolerance under controlled environment in the National Phytotron Facility at ICAR-IARI, New Delhi and under screen house conditions at ICAR-Central Soil Salinity Research Institute, Karnal for two seasons namely, *Kharif* (Jun-Sep) and *Rabi* (Nov-Mar). In the phytotron, the ambient temperature was regulated from 30–35°C during the day and 20–24°C during night with a relative humidity in the range of 70–80%. Pre-germinated (3 days after germination) seeds were sown in wells punched on extruded polystyrene foam floats fitted with a nylon wire mesh on the bottom side and fixed on plastic crates. Each crate was filled with 10 l of Yoshida nutrient solution (Yoshida et al., 1976) and carried 10 selected lines along with the sensitive (PB1121) and highly tolerant (FL478) parents. There were two replications for each set of lines, and each line was having nine plants per replication. In order to avoid border effect, FL478 was sown all along the border covering all sides to normalize competition for light and space. Fourteen days after germination, salt stress was imposed by adding 60 mM NaCl (ECe of 6.9 dS/m) in the solution culture and the concentration of salt in the nutrient solution was increased to 120 mM (ECe of 13.9 dS/m) after 3 days which was maintained until final phenotypic scoring. The pH of 5.0 was maintained in the culture solution daily, with a replacement of entire nutrient solution in every 7 days. Sixteen days after imposing salt stress, the genotypes were visually scored using standard evaluation system for rice for salt stress symptoms (IRRI, 2013) as modified by Gregorio et al. (1997), with scores ranging from 1 (highly tolerant) to 9 (highly sensitive).

In the hydroponic screen, three randomly selected uniform looking plants per genotype from each replication were carefully extracted intact from the float wells and the whole plants were washed initially in tap water for 1 min followed by two distilled water washings. The plants were dried using lint free filter paper and the length of shoot and root were measured from the collar region by carefully stretching the plants over a stainless steel ruler. The plants were then cut at the collar region to separate root and shoot portions. For each plant, the fresh weight of shoot and root were immediately recorded. Further, the root and shoot samples were dried for 3 days at 60°C in a hot air oven and the dry weights were recorded. Dried samples of shoot and root were used for estimation of Na^+^ and K^+^ content in these tissues.

### Estimation of Salt Ion Concentrations in Shoot and Root

For determining the salt ion concentrations in shoot and root, known weight (about 1 g) of respective dried samples were ground well and mixed with 25 ml of 1N hydrochloric acid (HCl) in 30 ml test tubes and kept standing for 24 h (Yoshida et al., 1976). The mixture was shaken gently, and filtered through Whatman 1 filter paper and the solid fraction was discarded. 2 ml of the filtrate was diluted with 40 ml of 1N HCl in sealed polypropylene vials and the diluted extract was used to determine Na^+^, and K^+^ content in ELICO CL360 Flame photometer (Elico, Ltd, India). The ion concentration was computed using the formula,

$$Ion({Na}^{+}orK^{+})concentration=\frac{C\times d\times V}{1000\times DW}$$

where, C denotes the flame photometer reading, d the dilution factor and V the volume of extract(ml) and DW is dry weight of the sample (g). Standard curves were prepared separately for Na and K using KCl and NaCl solutions respectively, with serial dilutions ranging from 0 to 100 mg/l. Na^+^ and K^+^ content of shoot and roots were determined from the standard curve and expressed in mg/kg.

### Phenotypic Evaluation

The *Saltol* NILs (beyond BC_3_F_4_) along with two parents (PB1121 and FL478) were planted at a spacing of 20 cm × 15 cm in randomized complete block design for agronomic evaluation during *Kharif* 2012, 2013, and 2014 at the research farm of the Genetics Division, ICAR-Indian Agricultural Research Institute (ICAR-IARI), New Delhi. Data were recorded from five uniform looking plants at different crop stages for agro-morphological traits such as days to 50% flowering (DFF), plant height (PHT), number of tillers (NTL), panicle length (PNL), spikelet fertility (SPF), weight of 1000 grains (TGW) and per plant yield (YPP). The grain and cooking quality traits such as hulling and milling recovery (HUL and MIL respectively), kernel length before cooking (KLBC), kernel breadth before cooking (KBBC), kernel length-breadth ratio (KLBR), kernel length after cooking (KLAC), kernel breadth after cooking (KBAC), kernel elongation ratio on cooking (KERC), and aroma (AROM) were recorded as described in Joseph et al. (2004).

The agronomic, physiological and grain quality data from the improved salt tolerant lines of PB1121 was subjected to statistical analyses of variance and means (Gomez and Gomez, 1984). To establish associations among the parameters of ion homeostasis, Pearson’s correlation coefficients were worked out among root and shoot Na^+^ and K^+^ concentration, their proportions and salt tolerance score.

### Expression Profiling of OsHKT1;5 Gene

The expression of *OsHKT1;5* was analyzed in a set of four genotypes including two *Saltol* carrying NILs derived of PB1121 namely Pusa1734-8-3-3 (NIL3) and Pusa1734-8-3-26 (NIL26) and their parents PB1121 and FL478 in BC_3_F_4_ generation. These NILs had more than 94% of PB1121 genome recovery and had the phenotype similar to that of PB1121 together with consistent seedling stage salt tolerance at both in hydroponic and field conditions. The experimental setup for salt stress imposition was similar to that described above for screening seedlings for salt tolerance. Experiment was set up in the National Phytotron Facility ICAR-IARI, New Delhi. Two different levels of salt stress were imposed on 21st day after sowing by filling the trays with Yoshida nutrient solution containing 50 mM (moderate stress) and 100 mM (high stress) of NaCl, together with an unstressed control in three replicates that was maintained using normal Yoshida nutrient solution.

Total RNA was isolated from the shoots tissues of each genotype collected at 0, 3, 12, and 24 h after imposing stress in each replication and in each level of stress and control. The isolation of total RNA from all the samples was carried out using RNeasy plant mini kit (Qiagen) following manufacturer’s protocol. The RNA concentration of samples was determined using NanoDrop^TM^ 1000 spectrophotometer (ThermoFisher Scientific, USA). Before running on a 1% RNase free agarose gel with an RNA size marker (MBI Fermentas, USA), the quality of total RNA was checked by incubating 2 μl of each sample for 3 min at 85°C to denature RNA in order to identify if any degradation of RNA had occurred. The clear integrity of 18S and 28S ribosomal RNA bands was checked to determine the non-degradation of total RNA. cDNA was synthesized using the Affinity Script QPCR cDNA synthesis Kit (Agilent Technologies, USA) fully optimized for two step quantitative real-time PCR (qRT-PCR, Heid et al., 1996).

The expression of *OsHKT1:5* gene was tested using the primer sequences (F-TTCATGGCGGTCAACTCGA and R-TTTGCTGGTGTTTGTCTTGGA) (Walia et al., 2005) using qRT-PCR, with 18 sRNA (F-TGATAACTCGACGGATCGC, R-CTTGGATGTGGTAGCCGTTT) (Kim et al., 2003) used as endogenous control. 18 sRNAwas used since the levels of this molecule were found to be fairly stable throughout the stages of plant development. All the reactions were performed in a Stratagene Mx3005P qPCR system (Agilent technologies, USA) using KAPA SYBER FAST qPCR master mix (2x) universal (KAPA Biosystems, USA^®^) for real time amplification. Each PCR reaction was performed in six replications and the amplification results were analyzed using Stratagene Mx3005P software (Agilent Technologies, USA). Relative quantitative expression was calculated based on log fold change (dRn) compared with respective controls (Livak and Schmittgen, 2001).

## Results

### Marker Assisted Introgression of *Saltol*

Parental polymorphism for the markers flanking the *Saltol* QTL identified RM8094 (11.2 Mb) and RM493 (12.3 Mb) as the nearest flanking polymorphic markers, which could be used for recombinant selection. In order to transfer *Saltol*, 10 BC_1_F_1_ plants heterozygous for the *Saltol* linked marker RM3412 were selected from 20 BC_1_F_1_ plants generated by backcrossing a single F_1_ plant to the recurrent parent PB1121 (**Table 1**). Out of 600 genome-wide STMS markers, 90 were polymorphic between PB1121 and FL478, that were then used for the background recovery analysis among the *Saltol* positive plants to ensure maximum RPG recovery in each backcross generation together with an additional set of 11 polymorphic markers specific to chromosome 1 for assessment of the introgression of the target QTL region. The recovery of RPG among the *Saltol* heterozygotes ranged between 76.0 and 84.4%. The BC_1_F_1_ plant with the highest RPG recovery of 84.4% as well as agro-morphological similarity with PB1121 was backcrossed to PB1121 to generate 15 BC_2_F_1_ plants. Foreground selection in BC_2_F_1_ revealed that seven plants heterozygous for *Saltol* which were further subjected to background selection using 28 markers heterozygous in the selected BC_1_F_1_ plant. Two BC_2_F_1_ plants were identified based on the morphological similarity to PB1121, which had an RPG recovery of 89.5 and 94.2% and least donor segment as determined by the recombinant selection using two polymorphic markers flanking *Saltol* locus. Twenty BC_3_F_1_ plants were produced from the two selected BC_2_F_1_ plants by another round of backcrossing. These BC_3_F_1_ plants were subjected to foreground, background, recombinant and morphological selection to identify three BC_3_F_1_ plants heterozygous for *Saltol* with an RPG more than 96%, which were selfed to produce BC_3_F_2_ populations. Fifty-one plants identified as homozygous for the foreground marker RM3412 were selected from BC_3_F_2_population from the initially short listed 198 plants based on similarity to PB1121 for morphological and grain quality characteristics. The number of selected lines were further reduced to 23 plants at the end of BC_3_F_3_ generation after a hydroponic screening for salt tolerance. Advancement of the selected 23 lines to successive generations by agronomic evaluation for three consecutive seasons resulted in selection of four superior plants with Basmati characters, namely Pusa 1734-8-3-3 (NIL3), Pusa 1734-8-3-26 (NIL26), Pusa 1734-8-3-30 (NIL30), and Pusa 1734-8-3-52 (NIL52).

**Table 1:** Backcross selections for marker assisted introgression of *Saltol* QTL in PB1121.

Generation	No. of plants generated	No. of *Saltol* positives^†^	No. of selected plants	Genome recovery (%)
F_1_	24	22	1	^∗^
BC_1_F_1_	20	10	1	76.0–84.0
BC_2_F_1_	15	7	2	89.5–94.2
BC_3_F_1_	20	9	3	>96.0
BC_3_F_2_	198	51	51	^∗^
BC_3_F_3_	51	51	23	^∗^
BC_3_F_4_+	23	23	4	93.3–99.0


*^∗^Not estimated; ^†^*Saltol* positives indicate the number of plants that were found to carry the target foreground marker alleles for *Saltol* QTL. BC_*3*_F_*4*_+, Generations BC_*3*_F_*4*_ and beyond on selfing.*

**FIGURE 1:**
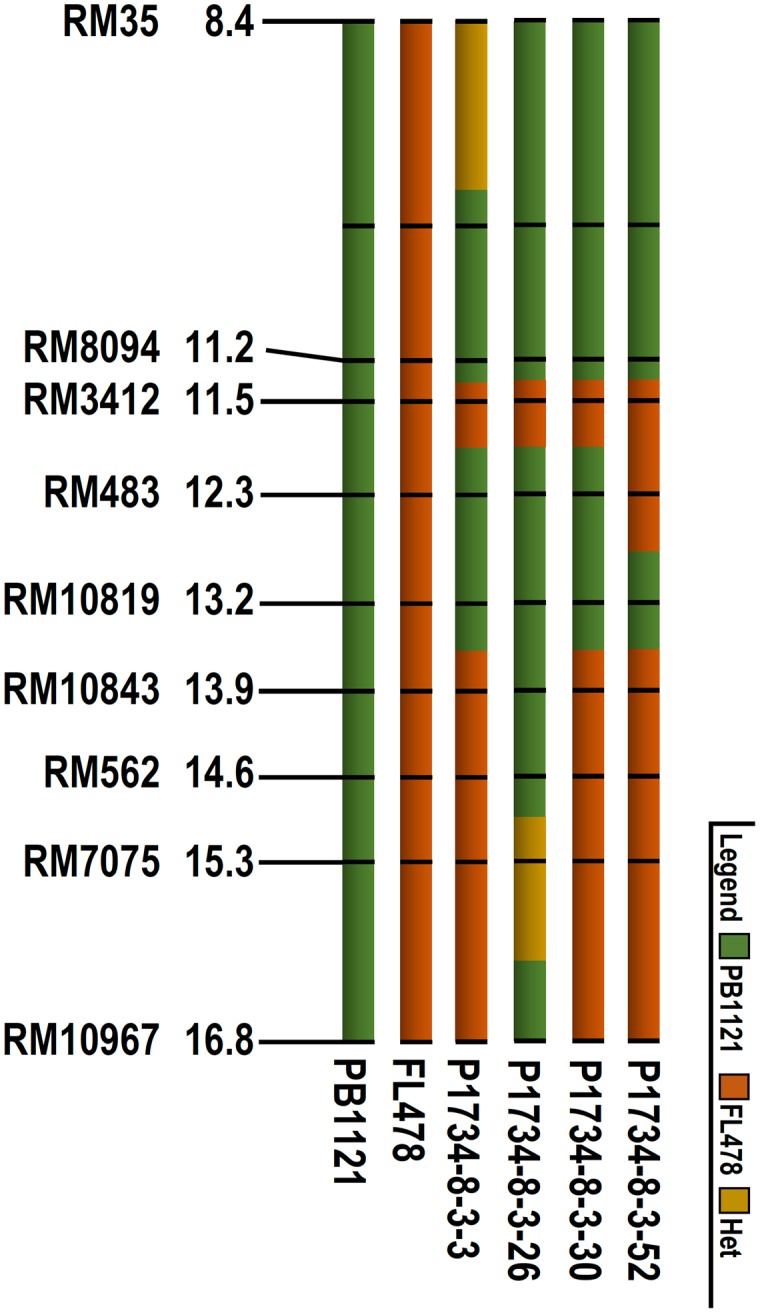
**Graphical genotype comparison of the *Saltol* region of parental lines (PB1121 and FL478) and the NILs.** The extent of donor segment introgression was determined by respective flanking marker alleles.

Further, additional polymorphic markers were used to assess the genomic contributions on carrier chromosome of the selected homozygous lines, through which one *Saltol* homozygous genotype NIL26 with less than 0.3 Mb donor segment in *Saltol* region (**Figure 1**) and with 99.4% background genome recovery could be identified. The RPG recovery in the other NILs ranged from 93.3 (NIL 30) to 94.4 (NIL3) (**Table 3**).

### Screening for Seedling Stage Salinity Tolerance

Fifty-one BC_3_F_3_ *Saltol* homozygous lines were screened in hydroponics along with parents, PB1121, FL478 and a susceptible check IR29 at New Delhi and Karnal. The salt tolerance response of the lines at both locations were similar. IR29 was highly susceptible to salt stress with a score of 9, while 18 *Saltol* homozygous lines were found to be tolerant with an average score of 3, the tolerant score similar to that of the donor parent FL478. Among the rest of the lines, 17 lines were found to be moderately tolerant with a score of 5 and 16 lines were susceptible with a score of 7. A set of 23 NILs including 18 NILs with score of 3 and 5 with score of 5 were selected for further evaluation of agronomic performance, grain and cooking quality.

### Field Level Agronomic Performance, Grain and Cooking Qualities of NILs

The field evaluation of agronomic performance with respect to yield and yield components were carried out with twenty-three *Saltol* homozygous lines for three consecutive rice growing seasons spanned over 3 years (**Supplementary Tables S1** and **S2**). Analysis of variance (**Table 2**) revealed significant effects of years and NILs for all the traits, except for panicle length, which had significance variation among NILs alone. The interaction component of NILs and Year was also significant for all traits but panicle length and 1000 grain weight. Four superior lines were identified based on salt tolerance, agronomic and grain quality evaluations and the data from the selected lines are presented in the **Tables 3** and **4**. With regard to yield and yield components, all the lines were found similar to PB1121 except for NIL3, which was slightly taller (142.4 cm) but had the advantage of shorter duration (DFF- 93.8 days). Among the remaining lines, PHT varied from 117.4 cm (NIL30) to 130.8 cm (NIL52) as against 120.6 cm in PB1121 and DFF ranged from 100.8 (NIL26) to 104.2 (NIL52) as compared to 106.2 days of PB1121.

**Table 2:** Variances of various agronomic traits by combined analysis of variance of the multi-year evaluation of *Saltol* NILs.

	PHT	PNL	NTL	DFF	SPF	TGW	YPP
Year	3766.6	ns	941.7	562.5	2571.6	298.1	3660.5
Rep (Year)	ns	ns	ns	ns	117.4	ns	149.9
NILs	533.6	8.3	26.5	46.3	480.3	9.4	131.9
Year × NILs	131.4	ns	10.8	24.6	80.6	ns	68.4
Mean^∗^	126.2	28.72	17.15	102.62	76.74	25.01	44.2
CV%	5.1	6.71	9.84	2.01	7.72	8.15	9.59


*^∗^Overall mean values in respective units for the agronomic traits; PHT, plant height in cm; PNL, panicle length in cm; NTL, number of tillers; DFF, days to 50% flowering; SPF, spikelet fertility in %, TGW, weight of 1000 grains in g; YPP, grain yield per plant in g; CV%, coefficient of variation in %; ns, non-significant at *p* < 0.05.*

**Table 3:** Agronomic performance and recurrent parent genome (RPG) recovery in improved lines in comparison to recurrent (PB1121) and donor (FL478) parents.

NIL	Genotype	PHT	NTL	PNL	DFF	SPF	TGW	YPP	RPG	STS
NIL3	Pusa 1734-8-3-3	142.4 a	15.9 a	29.6 a	93.8 b	84.3 a	27.6 a	41.3 a	94.4	3.0
NIL26	Pusa 1734-8-3-26	129.0 b	16.6 a	29.1 a	100.8 ab	86.6 a	23.1 a	48.5 a	99.4	3.0
NIL30	Pusa 1734-8-3-30	117.4 b	15.3 a	27.7 a	103.8 ab	81.3 a	25.0 a	39.6 a	93.3	3.0
NIL52	Pusa 1734-8-3-52	130.8 ab	16.4 a	28.9 a	104.2 a	75.4 a	24.4 a	41.7 a	93.9	3.0
-	PB1121 (recipient)	120.6 b	16.5 a	28.6 a	106.2 a	74.3 a	25.5 a	43.1 a	100.0	7.0
-	FL478 (donor)	97.0 c	10.7 b	25.7 a	88.0 b	86.5 a	23.2 a	28.4 b	0.0	3.0
	CD^†^	12.9	4.8	ns	10.0	ns	ns	10.2	-	-


*NIL, near isogenic line; PHT, plant height in cm; NTL, number of tillers; PNL, panicle length in cm; DFF, Days to 50% flowering; SPF, spikelet fertility in %, TGW, weight of 1000 grains in g; YPP, grain yield per plant in g; RPG, recurrent parent genome recovered expressed in %; STS, salt tolerance score (0–9 Scale): 1, highly tolerant; 3, tolerant; 5, moderately tolerant; 7, sensitive; 9, highly sensitive; ns, non-significant at *p* < 0.05.*

*^†^Pairwise critical difference by Tukey’s honestly significant difference (HSD) test; means followed by same letters are significantly not different at *p* < 0.05.*

**Table 4:** Grain dimensions and quality traits of improved lines in comparison to Pusa Basmati 1121.

NIL	Genotype	HUL	MIL	KLBC	KBBC	KLBR	KLAC	KBAC	KERC	ASV	AROM
NIL3	Pusa 1734-8-3-3	79.0 a	65.0 a	8.62 a	1.57 a	5.93 a	18.8 a	2.05 a	2.17 a	7.0 a	2.0 a
NIL26	Pusa 1734-8-3-26	78.1 a	66.0 a	8.41 a	1.42 a	6.36 a	18.4 ab	2.05 a	2.16 a	7.0 a	2.0 a
NIL30	Pusa 1734-8-3-30	78.8 a	64.6 a	8.24 a	1.46 a	5.64 a	18.8 a	1.95 a	2.04 a	7.0 a	2.0 a
NIL52	Pusa 1734-8-3-52	77.1 a	64.3 a	8.78 a	1.53 a	6.04 a	18.4 ab	2.11 a	2.10 a	7.0 a	2.0 a
-	PB1121 (recipient)	79.0 a	66.8 a	8.59 a	1.59 a	5.37 a	18.3 a	2.27 a	2.14 a	7.0 a	2.0 a
-	FL478 (donor)	79.4 a	64.3 a	6.24 b	2.36 b	2.65 b	10.2 b	3.69 b	1.63 b	5.0 b	0.0 b
	CD^†^	ns	ns	1.23	0.25	1.03	1.24	1.24	0.32	0.5	0.1


*HUL, hulling recovery in %; MIL, milling recovery in %; KLBC, kernel length before cooking in mm; KBBC, kernel breadth before cooking in mm; KLBR, length/breadth ratio; KLAC, kernel length after cooking in mm; KBAC, kernel breadth after cooking in mm; KERC, kernel elongation ratio on cooking; AROM, aroma score (0, unscented; 1, lightly scented; 2, scented; 3, highly scented); CD, critical difference; ns, non-significant at *p* < 0.05.*

*^†^Pairwise critical difference by Tukey’s honestly significant difference (HSD) test; means followed by same letters are significantly not different at *p* < 0.05.*

**FIGURE 2:**
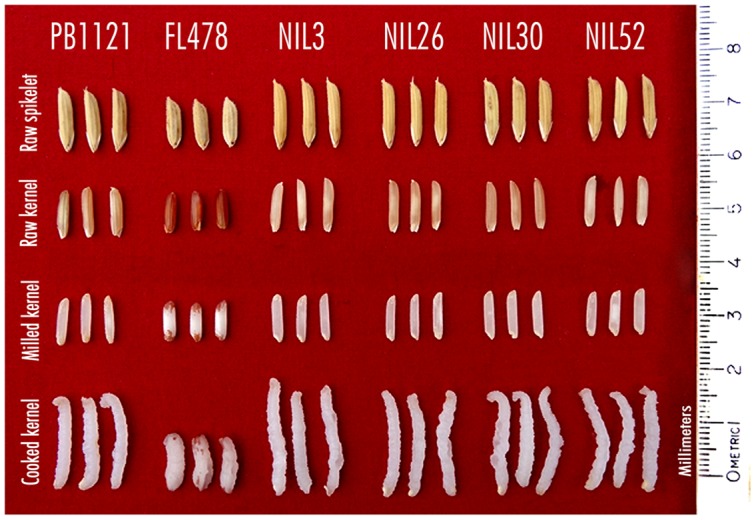
**Grain and cooking quality of parents (PB1121 and FL478) and advanced derived lines, Pusa 1734-8-3-3 (NIL3), Pusa 1734-8-3-26 (NIL26), Pusa 1734-8-3-30 (NIL30), and Pusa 1734-8-3-52 (NIL52)**.

All the improved NILs possessed extra-long (>8.0 mm length) and slender (length-width ratio of >5.0) grains (**Figure 2**) and other grain and cooking quality traits similar to that of the recurrent parent PB1121. The KLBC of the NILs ranged from 8.24 mm (NIL30) to 8.78 mm (NIL3) as compare to 8.59 mm in PB1121. The KLBR was in the range of 5.64 (NIL30) to 6.36 (NIL26) as compared with 5.37 of PB 1121. Similar to PB1121, all the NILs had superior KERC (≥2) as well as high aroma (**Table 4**).

### Analysis for Salt Ion Concentration in the Saltol NILs

The root and shoot of Na^+^ and K^+^ ionic concentration in the seedlings of the four superior *Saltol* NILs of PB1121, were assessed after subjecting them to salt stress (**Table 5**). Under salt stress, the donor and recurrent parents significantly differed in their ionic concentrations both in root and shoot. Na^+^ concentration in shoot (1.81 mmol/g of dry weight) and root (1.27 mmol/g of dry weight) of PB1121 under salt stress was 3- and 5-fold more than the donor parent FL478 (0.6 mmol/g of shoot dry weight and 0.24 mmol/g of root dry weight), respectively. The root Na^+^ concentration among the *Saltol* NILs showed varying results. NIL26 and NIL30 had higher Na^+^ concentration in root than FL478 but significantly lower that of PB1121, whereas NIL3 and NIL52 had equal or higher levels of root Na^+^ content than PB1121. Similar trend was also observed for root K^+^ concentration.

**Table 5:** Biochemical traits in the selected *Saltol* introgressed PB1121 NILs lines with respect to donor and recipient parents.

Code	Genotype	Root^†^			Shoot		
			
		Na^+^	K^+^	Na/K	Na^+^	K^+^	Na/K
NIL3	Pusa 1734-8-3-3	1.29 a	0.13 b	9.66 a	1.89 a	0.81 ab	2.34 ab
NIL26	Pusa 1734-8-3-26	0.52 b	0.46 a	1.13 b	1.02 b	0.67 b	1.51 b
NIL30	Pusa 1734-8-3-30	0.74 b	0.40 a	1.83 b	0.99 b	0.90 a	1.11 b
NIL52	Pusa 1734-8-3-52	1.65 a	0.20 b	8.16 a	2.83 a	0.78 b	3.64 a
-	PB1121 (recipient)	1.27 a	0.17 b	7.53 a	1.81 a	1.00 a	1.81 b
-	FL478 (donor)	0.24 c	0.41 a	0.59 b	0.60 b	0.59 b	1.01 b
	CD	0.40	0.10	2.20	1.10	0.20	1.40


*Na^+^, sodium ion concentration in mmol/g; K^+^, potassium concentration in mmol/g; Na/K, sodium–potassium ratio; CD, critical difference.*

*^†^Pairwise critical difference by Tukey’s honestly significant difference (HSD) test; Means followed by same letters are significantly not different at *p* < 0.05.*

K^+^ concentration in shoots of PB1121 (1.00 mmol/g of dry weight) was almost double the concentration of K^+^ in FL478 (0.59 mmol/g of dry weight). In *Saltol* NILs, shoot Na^+^ concentration ranged from 0.99 mmol/g of dry weight (NIL30) to 2.83 mmol/g of dry weight (NIL52). The Na^+^/K^+^ ratio in shoot of FL478 was 1.01 as against 1.81 of PB1121. The root Na^+^/K^+^ ratio in FL478 was 0.59 while PB1121 had almost 12 times higher Na^+^/K^+^ ratio (7.53). Among the introgressed lines, the shoot Na^+^/K^+^ ratio ranged from 1.11 (NIL30) to 3.64 (NIL52), while the lowest root Na^+^/K^+^ ratio was recorded 1.13 in NIL26 and the highest ratio of 9.66 in NIL3. The selected salinity tolerant advanced backcrossed lines like NIL30 and NIL26 had shoot Na^+^/K^+^ ratio lesser than PB1121 and NIL30 had ratio similar to FL478. However, other introgression lines (NIL3 and NIL52) recorded higher shoot Na^+^/K^+^ rates than PB1121, in spite of being tolerant to salt stress.

Interrelations between Na^+^ and K^+^ concentration of the *Saltol* NILs indicated that Na^+^ concentration in root was significantly and negatively associated with K^+^ concentration in root but had significantly positive correlation with Na^+^/K^+^ ratio in root and shoot Na^+^ concentration (**Table 6**; **Supplementary Figure S1**). K^+^ concentration in root exhibited significant negative correlation with root and shoot Na^+^/K^+^ ratio and Na^+^ concentration in shoot while it showed negative correlation with K^+^ in shoot (*r* = 0.54). Root Na^+^/K^+^ ratio showed significantly positive association with shoot Na^+^ (*r* = 0.66) and shoot Na^+^/K^+^ (*r* = 0.59). Shoot K^+^ concentration showed significant negative association with shoot Na^+^/K^+^ (*r* = -0.89). Salt tolerance score was poorly associated with all ionic concentration and their ratios in shoot and root except with root K^+^ concentration (**Supplementary Figure S2**).

**Table 6:** Correlation coefficients among ion parameters and salt tolerance.

	Root			Shoot		
		
	Na^+^	K^+^	Na/K	Na^+^	K^+^	Na/K
Root K^+^	-0.45^∗^					
Root Na^+^/K^+^	0.73^∗∗^	-0.83^∗∗^				
Shoot Na^+^	0.93^∗∗^	-0.49^∗^	0.66^∗∗^			
Shoot K^+^	0.12	0.54^∗∗^	-0.41	0.07		
Shoot Na/K	0.18	-0.68^∗∗^	0.59^∗∗^	0.26	-0.86^∗∗^	
STS	-0.11	-0.49^∗^	0.29	-0.10	-0.17	0.06


*Na^+^, sodium ion concentration in mmol/g; K^+^, potassium concentration in mmol/g; Na/K, sodium–potassium ratio; STS, salt tolerance score.*

*^∗^,^∗∗^Critical values of two tailed test, *p* < 0.05 = 0.423 and *p* < 0.01 = 0.537 at df = 20.*

### Expression Profiling of OsHKT1;5 Gene

The quantitation curves of the *OsHKT1;5* mRNA isolated from four genotypes (FL478, PB1121, NIL3, and NIL26) showed that the PCR amplification proceeded normally. The melting curves obtained indicated that one specific product was generated in each PCR reaction. The general observation was that mean relative quantity of *OsHKT1;5* was very low in shoots at early seedling stage.

**FIGURE 3:**
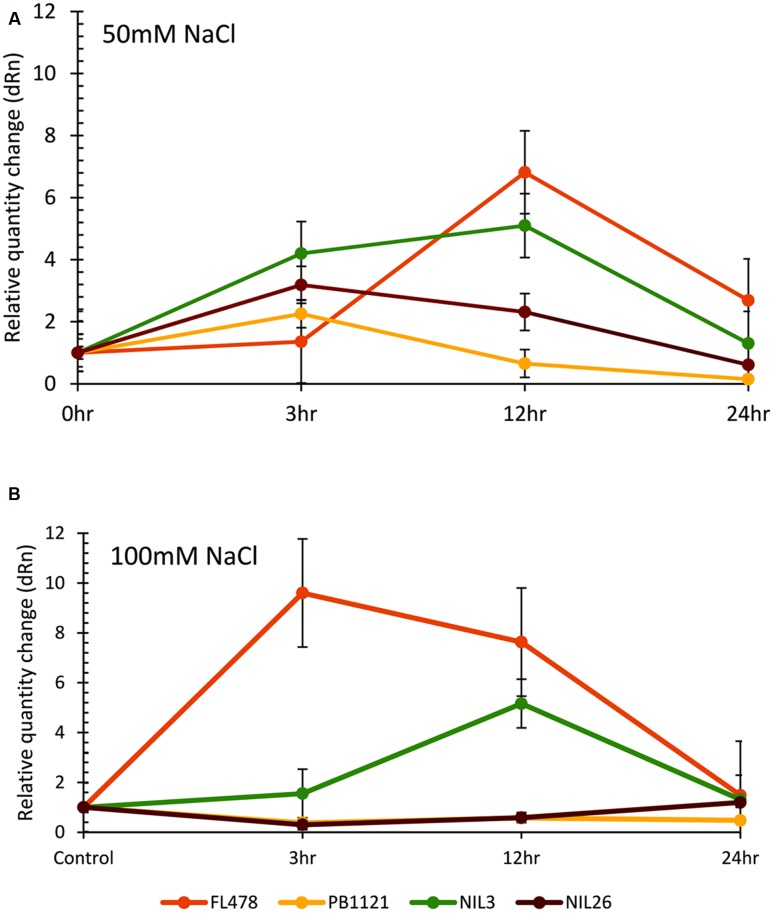
**Relative expression of *OsHKT1:5* gene at different time intervals (hours) observed in seedlings of parents (PB1121 and FL478) and two NILs (NIL3 and NIL26) after 50 mM** **(A)** and 100 mM **(B)** salt stress exposure.

Upregulation of *OsHKT1;5* was observed in the salt tolerant NILs of PB1121 immediately after salt exposure under moderate stress (50 mM), while under severe stress (100 mM) PB1121 and NIL26 failed to show any significant change (**Figure 3**). FL478 had the highest level of *OsHKT1;5* expression under salt exposure both under high and moderate levels, as compared to all other lines. Under moderate stress, all the NILs showed higher level *OsHKT1;5* gene expression than PB1121. *OsHKT1;5* mRNA expression (**Figure 3A**) in the seedling shoots of PB1121 and NIL26 was elevated from 0 to 3 h and decreased further as time advanced. In the NILs, where upregulation of *OsHKT1;5* was observed, the peaking followed a gradual reduction along the period of exposure. However, in FL478 and in NIL3, *OsHKT1;5* mRNA expression increased from 0 to 12 h and then declined further. The relative quantity change (fold change) under 50 mM salt treatment showed that *OsHKT1;5* expression level in NIL3 and FL478 were upregulated more than 3- to 4-fold 3 h after salt exposure as compare to unstressed control, while in PB1121 and NIL26 only less than 2- to 3-fold increase was observed.

Under high (100 mM) salt concentrations, almost a similar trend was observed as that of under moderate stress, it was interesting to note a distinct difference in the degree of *OsHKT1;5* expression levels among the lines, in which FL478 had a slower response to moderate stress, which changed to a rapid response under severe stress, while the degree of expression was much lower in NIL26 and PB1121. Whereas, NIL3 showed an intermediate type of expression. In NIL3, *OsHKT1;5* expression was distinctly higher from that of NIL26 but lower than FL478. However, in NIL26 *OsHKT1;5* expression was almost comparable as that of PB1121 up to 12 h of exposure beyond which it started to increase, indicating a recovery beyond 12 h of stress exposure (**Figure 3B**). The mRNA level of *OsHKT1;5* in seedling shoots of FL478 increased more than ninefold after exposure for 3 h as compare to control, while in NIL26 observed increase was just below twofold after 3 h of exposure. However, the mRNA expression increased in NIL26 to more than fivefold after 12 h that declined as the time progressed. Whereas in the case of FL478, expression level declined to sevenfold level further declining steadily until 24 h of stress exposure. In general, expression of mRNA of *OsHKT1;5* in shoot of FL478 is more in high stress than under moderate stress, while PB1121 and NIL26 showed lower level of expression in high stress than moderate stress. The mRNA expression level of NIL3 was comparable under both the stress treatments.

## Discussion

Basmati rice, especially the most popular Basmati rice variety PB1121, grown in the GI region of India, suffers from soil salinization, resulting in significant yield loss due to poor crop establishment as well as poor yield incited by the soil salinity. Incorporating QTLs such as *Saltol* identified for imparting seedling stage salt tolerance into PB1121 through marker assisted backcrossing (MAB) can help in alleviating the problem due to soil salinity in Basmati rice. FL478 has been extensively used as the donor for *Saltol* into elite varieties through MAB (Yeo and Flowers, 1986; Rahman et al., 2008; Thomson et al., 2010; Hien et al., 2012; Huyen et al., 2012; Guo and Ye, 2014). MAB offers remarkable advantage over conventional selection in improving genotypes for various traits due its high efficiency and simplicity and has been successfully demonstrated in developing improved rice varieties with inbuilt resistance to various biotic and abiotic stresses such as bacterial blight (Huang et al., 1997; Chen et al., 2000, 2001; Joseph et al., 2004; Zhang et al., 2006; Gopalakrishnan et al., 2008; Sundaram et al., 2008, 2009; Basavaraj et al., 2009, 2010; Bhatia et al., 2011; Singh A. et al., 2012; Singh et al., 2013), blast (Hittalmani et al., 2000; Zhou et al., 2011; Singh V.K. et al., 2012; Singh et al., 2013), submergence tolerance (Neeraja et al., 2007; Jantaboon et al., 2011) and salinity tolerance (Rahman et al., 2008; Huyen et al., 2012). Advantages and potentialities of modifications of MAB like foreground, background and recombinant selection for improvement of Basmati rice varieties were established by many previous works (Joseph et al., 2004; Gopalakrishnan et al., 2008; Basavaraj et al., 2010; Singh A. et al., 2012; Singh V.K. et al., 2012; Singh et al., 2013). MAB had brought in a paradigm shift in Basmati breeding (Singh et al., 2011), because foreground selection for the target trait was driven by marker genetics, while parallel stringent phenotypic selection augmented marker based background selection helped to retain key Basmati quality traits to the selected lines thus hastening the entire breeding process. The present work is a successful demonstration of MAB supplemented with phenotypic selection for agro-morphological, grain and cooking quality traits for introgression of *Saltol*, a major QTL for seedling stage salt tolerance into an elite Basmati rice variety, PB1121.

All the selected lines, possessed yield levels similar to that of the recurrent parent PB1121, along with Basmati grain and cooking quality characters. The RPG recovery response obtained in the selected lines to the tune of 93.3–99.4% categorically established the advantage of marker based background selection augmented with stringency of phenotype selection (Singh et al., 2013; Ellur et al., 2016b). This approach had not only helped in precise introgression of *Saltol* locus with reduced linkage drag but helped also in keeping all Basmati grain and cooking quality traits intact, through just three backcrosses, despite the donor FL478 being a non-Basmati genotype with very contrasting grain and cooking qualities such as red pericarp (Babu et al., 2014), bold kernels and non-aromatic. Three backcrosses were resorted to recover the grain quality traits completely. In the present study, some of the selected lines were slightly different from the recurrent parent in terms of reduced duration and/or increased plant height, which was possible due to phenotypic selection, while keeping the Basmati quality intact. Improvement of recurrent parent with respect to non-target trait has already been demonstrated in MAB scheme with phenotype augmented marker based background selection (Gopalakrishnan et al., 2008; Singh et al., 2011).

During phenotypic screening for seedling stage salinity response, some of the *Saltol* NILs with high level of RPG recovery, showed varied levels of salt tolerance. This deviation was not unexpected, since salinity tolerance by itself is a quantitative trait under high environmental influence. Furthermore, since *Saltol* locus spans over a large genomic region (<1 Mb) and possibly consists of many candidate genes including *OsHKT1;5* (Ren et al., 2005; Walia et al., 2005), recombination within *Saltol* region can also be expected as a possible reason for differential level of salt tolerance among RM3412 positive lines. Besides, minor genetic background differences among the NILs cannot be ruled out because the background selection was carried out using a limited set of polymorphic markers between both the parents. It is pertinent to mention here that *Saltol* locus of FL478 is well-known to be influenced by background interactions between IR29 and Pokkali loci (Walia et al., 2005) that contained several Pokkali QTLs including that of *OsHKT1;5* (Thomson et al., 2010). Furthermore, chromosome 1 of FL478 had a Pokkali chromosomal fragment of <1 Mb size located at 10.6–11.5 Mb and flanked by IR29 fragments (Kim et al., 2009). Interaction between the target QTL/gene with the genetic background originating from common pedigree has also been reported earlier (Koide et al., 2011; Singh V.K. et al., 2012; Singh et al., 2013). Therefore, the differences observed in the level of salt tolerance at seedling stage among advanced *Saltol* introgressed families may be attributed to interactions of introgressed regions with genetic background that differed between lines and also to the recombinations within *Saltol* region, which needs further elucidation with in depth analysis.

Salt tolerance of rice is a manifestation of several components related to Na^+^ and K^+^ homeostasis. Physicochemical analysis of selected salt tolerant lines for shoot and root Na^+^ and K^+^ concentration and their ratio showed significantly different ionic concentrations (Vinod et al., 2013). Interrelations between Na^+^ and K^+^ content of the *Saltol* carriers including the improved lines and the donor parent FL478, showed that root and shoot Na^+^ concentration and root and shoot K^+^ concentration were significantly associated, possibly due to the sharing of common ion porting channels, by K^+^ and Na^+^ ions (Volkov, 2015). K^+^ uptake and translocation is high influenced by Na^+^ ions. On the other hand, root Na^+^ concentration had a negative impact on K^+^ concentration implying that competition existed in ion uptake in roots (Epstein, 1966), while in shoot Na^+^ and K^+^ concentrations were poorly related. Further, the root Na^+^/K^+^ ratio was weakly associated to salt tolerance score, but there was hardly any association between these traits in shoots. Although, earlier reports show that *Saltol* offers salt tolerance mainly by maintaining low Na^+^/K^+^ concentration in shoot (Gregorio, 1997; Bonilla et al., 2002), in the present work the shoot K^+^ content was found to show a significant negative relation to salt tolerance score indicating that higher shoot K^+^ offered better tolerance in the *Saltol* carriers. Maintaining shoot K^+^ concentration offered relative salt tolerance in *Saltol* carriers including FL478 (Hoang et al., 2016), although Na^+^ loading from root to shoots were different among different lines. Salinity tolerance in rice is not governed by a single mechanism, rather a combination of different mechanisms targeting low Na^+^/K^+^ ratio, Na^+^ sequestration, tissue tolerance, osmotic adjustment and extrusion (Asch et al., 1999, 2000; Zeng et al., 2003; Vinod et al., 2013). In most of the plants, the main site of Na^+^ toxicity is in the shoot rather than roots and therefore shoot Na^+^ concentration and its relation with K^+^ is more important in determining the salt tolerance (Tester and Davenport, 2003; Munns and Tester, 2008). Under saline conditions, accumulation of Na^+^ in shoots causes ionic imbalance particularly K^+^, which is vital for plant growth and development (Esechie et al., 2002; Munns, 2002). In the present observations, although root and shoot Na^+^ loadings showed positive relations, corresponding loading of shoot K^+^ content was significantly lower indicating existence of alternate mechanisms of Na^+^ sequestration and/or antiporting at root-shoot interphase (Pardo et al., 2006). Therefore, a genotype which has an ability to maintain low Na^+^/K^+^ ratio in shoots by mechanisms that preferentially load relatively more K^+^ than Na^+^ would be superior for salt tolerance (Vinod et al., 2013). Notwithstanding this observation, some of the *Saltol* introgression lines in the present study showed increased Na^+^/K^+^ ratio implying that the sequestered Na^+^ ions in shoot cell vacuoles might have contributed to a relatively high estimate of shoot Na^+^ content when all the cells are macerated for total Na^+^ estimation than the actual Na^+^ ions that are functionally available (Maathuis et al., 2014). Else, a different salt tolerant mechanism may be operational driven by the genomic complementation of the parents, warranting in depth analysis for deciphering the mechanism of salt tolerance in the *Saltol* introgressed lines.

Although, *OsHKT1;5* was originally identified as the gene underlying the *qSKC1* QTL responsible for maintaining high shoot K^+^ under salt stress in rice (Lin et al., 2004), the pattern of shoot expression of *OsHKT1;5* and its association to *Saltol* and its expression in early seedling stage under salt stress is not yet fully understood. Walia et al. (2005) reported that under salinity stress *OsHKT1;5* was induced in shoot tissues in both the sensitive (IR29) and tolerant (FL478) plants during early vegetative stage, but the induction under stress was significantly higher in FL478 than IR29. No significant upregulation of *OsHKT1;5* was observed in the shoots of FL478 and IR29 at panicle initiation stage (Walia et al., 2007). In another study, Ren et al. (2005) reported upregulation of *OsHKT1;5* in root and not in shoot tissues when challenged by salt stress. They suggested that *OsHKT1;5* gene could be playing a role in recirculation of Na^+^ ions for in conferring salt tolerance. Notwithstanding significant deviation in few genotypes, recently, Platten et al. (2013) reported a strong quantitative relation between leaf Na^+^ content and salt tolerance in an extremely divergent germplasm, including cultivated and wild *O. glaberrima* accessions.

We have used two salt concentration levels (50 and 100 mM) to examine the *OsHKT1;5* expression, because, in cereals, salt concentrations in the range of 50–100 mM is intermediate to osmotic stress and shock (Shavrukov, 2013). In general, the level of expression of *OsHKT1;5* in the NILs were intermediate to FL478 and PB1121, in both the salt treatments, except for NIL26 under 100 mM concentration. Salt concentration of 50 mM or less has been reported to be ideal for inducing salt stress in seedlings, without inciting root cell plasmolysis (Munns, 2002), which could further be progressed up to 100 mM, beyond which osmotic shock took over the stress response. Since the prime objective of the expression patterning was to validate the *Saltol* integration in the NILs, we had chosen shoot tissues, because there were several earlier reports of signifying conspicuous *OsHKT1;5* expression in shoot tissues under salt stress (Walia et al., 2005; Platten et al., 2013).

The NIL3 in this study had distinctly higher expression of *OsHKT1;5* than PB1121, and a low degree of variation in NIL26, implying that *Saltol* integration was successful in NILs. It also further evidenced that *OsHKT1;5* was indeed an associated gene of the *Saltol* QTL (Ren et al., 2005; Ismail and Thomson, 2011), albeit the pattern of variation differed temporally and quantitatively, suggesting the role of either background genome interaction or the allelic variants of *OsHKT1;5* contributed by the parents. *OsHKT1;5* was reported to have several allelic variants in rice gene pool, of which seven major *O. sativa* alleles had been identified and the most common type, the *Aromatic* allele was the most effective in Na^+^ exclusion (Platten et al., 2013). While Pokkali and other salt tolerant landraces such as Nona Bokra and Cheruvirippu carried this allele, FL478 seemed to have a less effective *Aus* allele. This work therefore provided potential leads, which warrants in depth investigations on the factors responsible for the fluctuations. These observations further tempted us to postulate that *OsHKT1;5* expression might not be the only key gene imparting salt tolerance at different salt concentrations (Kim et al., 2009; Cheng et al., 2012). Therefore, for successful development of salt tolerant rice cultivars, it is critical to evaluate the salinity in the target environments, QTL-background interactions and growth stage specific tolerance mechanisms, especially at seedling and reproductive stages (Platten et al., 2013).

Since saline conditions are characterized with high level of Na^+^ and low K^+^ excess Na^+^ can cause toxicity in rice seedlings (Flowers and Yeo, 1981; Yeo and Flowers, 1986). To mitigate Na^+^ toxicity, initial upregulation of *OsHKT1;5* expression is very important to maintain K^+^/Na^+^ balance in tolerant genotypes. In susceptible genotypes due to low level of *OsHKT1;5* expression, K^+^/Na^+^ balance is disrupted exposing the cells to excess Na^+^ resulting in toxicity and finally death (Hauser and Horie, 2010). When compared to NILs, *OsHKT1;5* expression was higher for stressed plants of FL478, indicating that ion homeostasis in FL478 is distinctly better resulting in greater salt tolerance even at higher salt concentrations such as 100 mM of NaCl (Senadheera et al., 2009). The intolerant PB1121 showed relatively lower level of *OsHKT1;5* expression under both the stress levels, especially under high stress, indicating that native *OsHKT1;5* of PB1121 may be effective only up to moderate salt levels beyond which it may fail. Perhaps, transferring *Aromatic* allele into PB1121 may bring in better salt tolerance expression then the *Aus* allele, which FL478 has contributed (Platten et al., 2013) in the present MAS program. This is may be true for NIL26 also, which had a delayed expression of *OsHKT1;5*, but could withstand higher salt concentrations up to 120 mM in the earlier screening. Differential regulations including temporal variations of transporter genes such as *OsHKT1*, *OsHKT2* and *OsVHA* (vacuolar H^+^-ATPase) which play a synergistic role in maintaining the ion homeostasis, under NaCl dominated stress are reported in rice genotypes varying in salt tolerance (Kader et al., 2006). Although, higher concentrations of 120 mM NaCl may be ideal for screening salt tolerant genotypes for their efficiency (Platten et al., 2013), the results of this work indicated that moderate level of stress (50 mM) was the best to investigate *OsHKT1;5* gene expression at early seedling stage, when the population had differential salt tolerance.

## Conclusion

This work reports the first ever successful marker assisted transfer of the *Saltol* QTL into a Basmati rice cultivar, PB1121 for conferring seedling stage salt tolerance using MAB combined with stringent phenotype selection for agro-morphological, grain and cooking quality traits. All the selected NILs possessed salt tolerance on par with the donor FL478, together with desirable Basmati grain and cooking quality traits and yield traits similar to PB1121. Physiological basis for salt tolerance in the improved *Saltol* NILs indicated preferential K^+^ loading into shoot tissues as the major mechanism for conferring tolerance, although other mechanisms to sequester or antiport Na^+^ ions may also be operational.

This also is a first attempt to associate *OsHKT1;5* gene expression to *Saltol* QTL using *Saltol* derived NILs in rice. Our results clearly implied that *OsHKT1;5* was one of the key candidate genes associated with seedling stage salinity tolerance in rice. However, the expression of *OsHKT1;5* was highly influenced either by allelic variations or by the background genome interaction, or both, as observed from the differential expression pattern among the parents and NILs at different salt exposure levels. This warrants in depth studies on the allelic pattern of *OsHKT1;5*, and their quantitative and temporal expression profiles under varying salt concentrations. Furthermore, the selection of donors with right kind of target allele, and also the recipient with desired background genome complementation may be key to successful in transfer of the *Saltol* QTL while breeding for salt tolerance in rice. Besides, it is important to characterize the target locations for salinity levels before choosing the breeding sources. Therefore, validation of the salt tolerance of several NILs derived from the *Saltol* transfer program to a new background is imperative in order to select best lines possessing desired trait combinations. In the present study, Pusa1734-8-3-3 (NIL3) showed *OsHKT1;5* expression closer to that of tolerant FL478 under salt stress and also exhibited field level tolerance to salinity at seedling stage. NIL3 was a good yielding line with good grain and cooking qualities similar to PB1121, together with the advantage of reduced duration. These high yielding, salt tolerant improved PB1121 lines will be evaluated in multi-location trials, especially at locations affected by inland salinity, before release to farmers for commercial cultivation as improved Basmati varieties. When compared to susceptible cultivars, improved NILs will have better seedling survival under saline prone areas, ultimately bringing the yield level up by better crop establishment. Additionally, these NILs can serve as valuable donors for salt tolerance in future Basmati breeding programs.

## Author Contributions

AKS and KVP conceptualized and designed the experiment, NNB, SGK, KKV, and MN conducted the experiments, NNB, VKS, RKE, and HB did field evaluation, NNB and SLK conducted hydroponic screening, NNB, MPS, and NKS carried out biochemical analysis and NNB, VR, RS, and AKY conducted RNA isolation and RT-PCR for expression profiling studies, NNB, KKV, SGK, AKS analyzed the data and prepared the manuscript. All the authors have read and approved the final manuscript.

## Conflict of Interest Statement

The authors declare that the research was conducted in the absence of any commercial or financial relationships that could be construed as a potential conflict of interest.
