# Supplementary material for: Marker Aided Incorporation of Saltol, a Major QTL Associated with Seedling Stage Salt Tolerance, into Oryza sativa ‘Pusa Basmati 1121’
Source: Front Plant Sci. 2017 Jan 26;8:41. doi: 10.3389/fpls.2017.00041 (PMC5266695; doi:10.3389/fpls.2017.00041)
Supplement: Supplementary file 1 [file Table_1.PDF]

Supplementary Table S1. Mean performance of *Saltol* introgressed PB1121 derived NILs for agronomic traits and salt tolerance score in comparison with the recipient parent, PB1121

NIL	Code	PHT	PNL	TLN	DFF	SPF	TGW	YPP	STS
PB1121		120.6 abcd	28.6 ab	16.5 a	106.2 a	74.3 a	25.5 a	43.1 a	7.0 a
Pusa 1734-8-3-3	NIL 3	142.4 a	29.6 ab	15.9 a	93.8 b	84.3 a	27.6 a	41.3 a	3.0 b
Pusa 1734-8-3-4	NIL 4	133.4 ab	26.1 b	18.5 a	101.0 ab	75.6 a	24.8 a	45.3 a	5.0 b
Pusa 1734-8-3-10	NIL 10	132.9 ab	29.3 ab	17.6 a	100.3 ab	79.4 a	26.7 a	45.6 a	5.0 b
Pusa 1734-8-3-17	NIL 17	130.9 abc	29.7 ab	15.9 a	100.8 ab	82.1 a	25.5 a	50.2 a	5.0 b
Pusa 1734-8-3-21	NIL 21	136.0 ab	28.9 ab	14.3 a	101.0 ab	76.5 a	24.9 a	46.6 a	5.0 b
Pusa 1734-8-3-23	NIL 23	128.5 abc	27.6 ab	15.9 a	103.8 a	78.3 a	24.8 a	41.1 a	5.0 b
Pusa 1734-8-3-25	NIL 25	136.0 ab	30.3 ab	17.9 a	99.8 ab	86.8 a	25.2 a	49.0 a	3.0 c
Pusa 1734-8-3-26	NIL 26	129.0 abc	29.1 ab	16.6 a	100.8 ab	86.6 a	23.1 a	48.5 a	3.0 b
Pusa 1734-8-3-29	NIL 29	134.8 ab	30.1 ab	15.8 a	106.5 a	74.8 a	24.2 a	35.4 a	5.0 b
Pusa 1734-8-3-30	NIL 30	117.4 abcd	27.7 ab	15.3 a	103.8 a	81.3 a	25.0 a	39.6 a	3.0 b
Pusa 1734-8-3-51	NIL 51	136.3 ab	31.1 a	15.7 a	104.0 a	84.7 a	25.0 a	43.3 a	5.0 b
Pusa 1734-8-3-52	NIL 52	130.8 abc	28.6 ab	16.4 a	104.2 a	75.4 a	24.4 a	41.7 a	3.0 b
Pusa 1734-8-3-55	NIL 55	118.4 abcd	26.9 ab	17.4 a	103.8 a	80.1 a	26.1 a	44.9 a	5.0 b
Pusa 1734-8-3-56	NIL 56	121.4 abcd	27.8 ab	20.4 a	103.3 ab	75.5 a	23.6 a	44.8 a	5.0 b
Pusa 1734-8-3-71	NIL 71	122.2 abcd	28.1 ab	18.0 a	104.7 a	78.8 a	26.3 a	44.0 a	5.0 b
Pusa 1734-8-3-75	NIL 75	120.9 abcd	28.6 ab	19.2 a	105.5 a	86.8 a	25.0 a	49.8 a	5.0 b
Pusa 1734-8-3-85	NIL 85	128.8 abc	29.1 ab	17.8 a	103.3 ab	72.7 ab	25.3 a	45.4 a	5.0 b
Pusa 1734-8-3-91	NIL 91	126.6 abcd	28.5 ab	18.9 a	103.8 a	83.7 a	27.7 a	49.4 a	5.0 b
Pusa 1734-8-3-97	NIL 97	125.9 abcd	28.7 ab	19.8 a	103.0 ab	79.5 a	24.8 a	49.3 a	5.0 b
Pusa 1734-8-3-176	NIL 176	130.1 abc	27.9 ab	20.0 a	103.3 ab	80.0 a	23.7 a	46.3 a	5.0 b
Pusa 1734-8-3-184	NIL 184	116.1 bcd	27.2 ab	17.7 a	98.2 ab	78.0 a	24.8 a	46.9 a	5.0 b
Pusa 1734-8-3-190	NIL 190	103.1 d	30.0 ab	13.2 a	104.3 a	52.9 b	22.7 a	32.0 a	5.0 b
Pusa 1734-8-3-191	NIL 191	107.1 cd	29.8 ab	14.9 a	103.5 ab	51.8 b	23.7 a	37.3 a	5.0 b
CD*		25.4	4.8	ns	10.0	21.0	ns	ns	0.1

* Pairwise critical difference by Tukey's honestly significant difference (HSD) test; Means with the same letter are not significantly different at p<0.5; ns, non-significant

DFF, Days to 50% flowering; PHT, plant height in cm; NTL, number of tillers; PNL, panicle length in cm; SPF, spikelet fertility in %, TGW, weight of 1000 grains in g; YPP, grain yield per plant in g; ns, non-significant at p<0.05
