# Supplementary material for: Marker Aided Incorporation of Saltol, a Major QTL Associated with Seedling Stage Salt Tolerance, into Oryza sativa ‘Pusa Basmati 1121’
Source: Front Plant Sci. 2017 Jan 26;8:41. doi: 10.3389/fpls.2017.00041 (PMC5266695; doi:10.3389/fpls.2017.00041)
Supplement: Supplementary file 2 [file Table_2.PDF]

Supplementary Table S2. Grain quality comparison of PB1121 derived NILs carrying *Saltol* QTL, with PB1121

NILs	Code	HUL	MIL	KLBC	KBBC	LBR	KLAC	KBAC	KER	ASV	ARO
Pusa 1734-8-3-3	NIL 3	79.03 a	65.00 a	8.62 a	1.57 a	5.93 a	18.79 abc	2.05 a	2.17 a	7.0 a	2.0 a
Pusa 1734-8-3-4	NIL 4	78.73 a	67.68 a	8.47 a	1.48 a	5.95 a	17.39 a	2.05 a	2.05 a	7.0 a	2.0 a
Pusa 1734-8-3-10	NIL 10	79.85 a	66.92 a	8.39 ab	1.38 a	6.10 a	17.12 a	2.14 a	2.04 a	7.0 a	2.0 a
Pusa 1734-8-3-17	NIL 17	78.73 a	68.08 a	8.15 ab	1.48 a	5.64 a	16.96 a	2.03 a	2.09 a	7.0 a	2.0 a
Pusa 1734-8-3-21	NIL 21	78.78 a	66.58 a	8.19 ab	1.46 a	5.91 a	17.13 a	1.97 a	2.09 a	7.0 a	2.0 a
Pusa 1734-8-3-23	NIL 23	79.30 a	66.28 a	8.37 ab	1.46 a	5.99 a	17.48 a	1.95 a	2.09 a	7.0 a	2.0 a
Pusa 1734-8-3-25	NIL 25	78.65 a	67.40 a	8.78 a	1.38 a	6.46 a	16.89 a	2.25 a	1.92 a	7.0 a	2.0 a
Pusa 1734-8-3-26	NIL 26	78.05 a	66.03 a	8.41 ab	1.42 a	6.36 a	18.44 ab	2.05 a	1.96 a	7.0 a	2.0 a
Pusa 1734-8-3-29	NIL 29	79.15 a	66.28 a	8.32 ab	1.58 a	5.32 a	16.00 abc	2.17 a	1.93 a	7.0 a	2.0 a
Pusa 1734-8-3-30	NIL 30	78.83 a	64.60 a	8.24 ab	1.46 a	5.64 a	18.83 a	1.95 a	2.04 a	7.0 a	2.0 a
Pusa 1734-8-3-51	NIL 51	78.25 a	66.03 a	8.62 a	1.44 a	5.99 a	16.81 a	1.99 a	1.95 a	7.0 a	2.0 a
Pusa 1734-8-3-52	NIL 52	77.13 a	64.30 a	8.78 a	1.53 a	6.04 a	18.35 ab	2.11 a	2.10 a	7.0 a	2.0 a
Pusa 1734-8-3-55	NIL 55	78.10 a	65.05 a	8.17 ab	1.45 a	5.64 a	16.73 a	1.93 a	2.06 a	7.0 a	2.0 a
Pusa 1734-8-3-56	NIL 56	79.43 a	65.83 a	8.67 a	1.45 a	6.04 a	16.55 a	2.05 a	1.91 a	7.0 a	2.0 a
Pusa 1734-8-3-71	NIL 71	78.38 a	65.78 a	8.84 a	1.54 a	6.00 a	17.27 a	2.00 a	1.95 a	7.0 a	2.0 a
Pusa 1734-8-3-75	NIL 75	77.10 a	64.98 a	8.32 ab	1.43 a	7.03 a	16.44 ab	2.04 a	1.98 a	7.0 a	2.0 a
Pusa 1734-8-3-85	NIL 85	78.33 a	67.12 a	8.80 a	1.46 a	6.38 a	17.38 a	2.09 a	1.98 a	7.0 a	2.0 a
Pusa 1734-8-3-91	NIL 91	77.48 a	66.28 a	8.65 a	1.47 a	5.90 a	17.85 a	2.09 a	2.06 a	7.0 a	2.0 a
Pusa 1734-8-3-97	NIL 97	77.73 a	65.48 a	8.66 a	1.46 a	6.50 a	16.98 a	2.18 a	1.96 a	7.0 a	2.0 a
Pusa 1734-8-3-176	NIL 176	78.48 a	66.92 a	8.46 a	1.50 a	5.78 a	15.89 abc	2.11 a	1.88 a	7.0 a	2.0 a
Pusa 1734-8-3-184	NIL 184	78.90 a	67.30 a	8.27 ab	1.54 a	5.47 a	16.53 ab	1.89 a	2.00 a	7.0 a	2.0 a
Pusa 1734-8-3-190	NIL 190	78.18 a	64.53 a	7.41 b	1.59 a	4.65 a	13.56 c	2.11 a	1.83 a	5.0 b	2.0 a
Pusa 1734-8-3-191	NIL 191	79.00 a	63.88 a	7.95 ab	1.58 a	5.50 a	13.93 bc	2.17 a	1.76 a	7.0 a	2.0 a
PB1121	-	79.03 a	66.77 a	8.59 a	1.59 a	5.37 a	18.30 a	2.27 a	2.14 a	7.0 a	2.0 a
CD*		ns	ns	1.03	ns	ns	2.61	ns	0.36	-	ns

* Pairwise critical difference by Tukey's honestly significant difference (HSD) test; Means with the same letter are not significantly different at $p<0.5$; ns, non-significant

HUL, hulling %; MIL, miling %; KLBC, kernel length before cooking (mm); KBBC, kernel breadth before cooking (mm); LBR, length-breadth ratio before cooking; KLAC, kernel length after cooking (mm); KBAC, kernel breadth after cooking (mm); KER, kernel elongation ratio; ASV, alkali spreading value; ARO, aroma
