# Supplementary material for: Marker Aided Incorporation of Saltol, a Major QTL Associated with Seedling Stage Salt Tolerance, into Oryza sativa ‘Pusa Basmati 1121’
Source: Front Plant Sci. 2017 Jan 26;8:41. doi: 10.3389/fpls.2017.00041 (PMC5266695; doi:10.3389/fpls.2017.00041)

Supplementary Fig 1. Interrelations of ionic concentration among *Saltol* introgressed lines and FL478

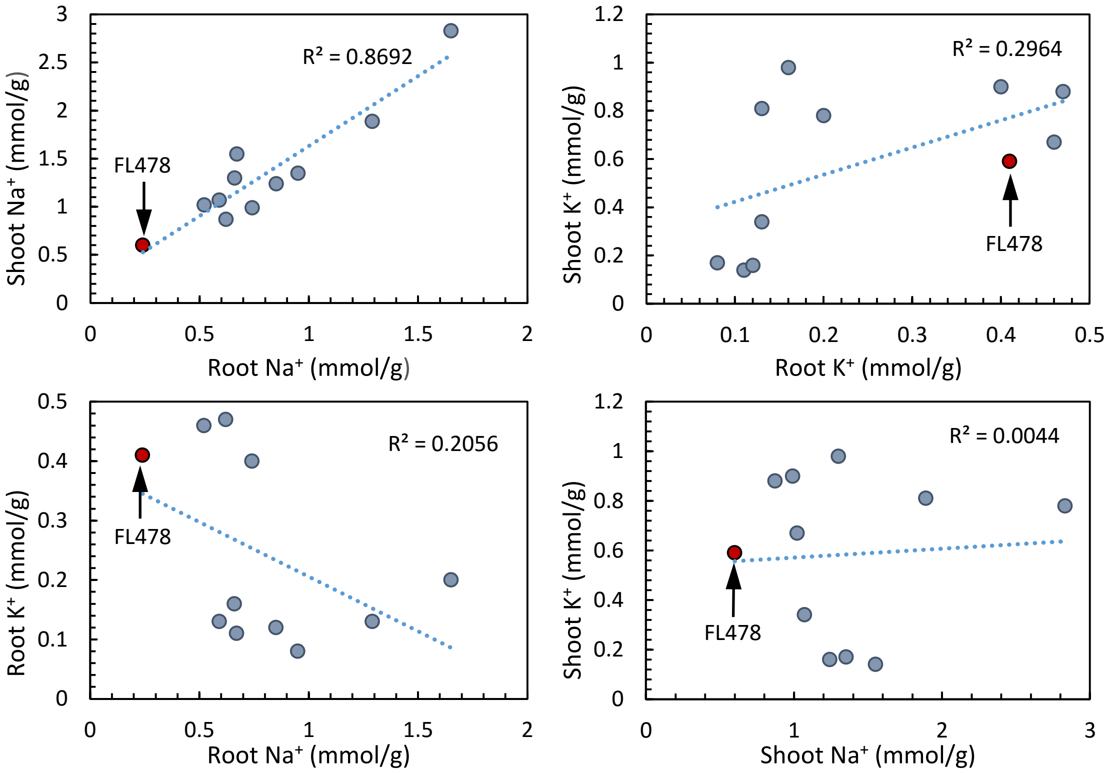

Supplement: Supplementary file 3 [file Image_1.PDF]
